# Robotic Partial Nephrectomy with Indocyanine Green Fluorescence Navigation

**DOI:** 10.1155/2020/1287530

**Published:** 2020-04-27

**Authors:** Lukas Gadus, Jiri Kocarek, Frantisek Chmelik, Marketa Matejkova, Jiri Heracek

**Affiliations:** ^1^Department of Urology, Military University Hospital, Prague 16902, Czech Republic; ^2^First Faculty of Medicine, Charles University, Prague 12108, Czech Republic; ^3^Department of Urology, First Faculty of Medicine, Charles University, Prague 12800, Czech Republic

## Abstract

Partial nephrectomy (PN) is a recommended type of treatment of localised renal tumors. Real-time intraoperative imaging technique, such as fluorescence imaging with indocyanine green (ICG) administration helps to improve intraoperative and postoperative outcomes in patients who underwent PN. Our work presents results of patients who underwent robotic PN with ICG navigation. A total of 37 patients underwent robotic PN with application of ICG between April 2015 and May 2019. A total amount of 5 mg of ICG was applied intravenously, and then robotic PN was performed with fluorescent imaging. ICG was used by the surgeon's decision according to unfavourable anatomical properties of tumor or to high R.E.N.A.L. nephrometry score. An exact border between perfused and nonperfused tissue was detected, and exact tumor's branch of the renal artery was clamped. Robotic PN with ICG-fluorescence imaging navigation was performed in 37 cases with a preoperative average diameter of tumor of 31 mm. The mean surgery time was 133 minutes, and the mean estimated blood loss was 190 mL. Arterial clamping was performed in 35 cases. The mean duration of warm ischemia was 14 minutes. Application of ICG enabled specific tumor-supplying vessel clamping in 25 cases. Two complications of grade II according to the Clavien-Dindo classification occurred intraoperatively, and one complication of grade III was observed. Renal function changes showed favourable results for the cases with superselective clamping. Finally, an administration of ICG eases superselective clamping of tumor-specific branch of renal artery and helps to preserve normal renal function with acceptable oncological results.

## 1. Introduction

Nephron-sparing surgery is a recommended type of treatment of localised renal tumors with a diameter ≤7 cm whenever it is technically possible. Simone et al. described oncological equivalence for partial nephrectomy and radical nephrectomy with 10-year progression for cT1 tumors and equivalent oncological outcomes for cT2 tumors [1]. Huang et al. described clear benefits of partial nephrectomy instead of radical nephrectomy: reduction of incidence of comorbidities and therefore improved long-term survival [2]. Main criteria for successful partial nephrectomy, defined as trifecta, are surgical quality (no intraoperative Clavien-Dindo ≥3 complications), cancer control (presented as negative surgical margins), and minimal loss of renal function [3]. Therefore, there are efforts of surgeons to use different real-time intraoperative imaging techniques to improve operative and postoperative outcomes in patients who underwent partial nephrectomy. Techniques such as intraoperative ultrasonography, fluorescence imaging, tumor-targeted dual-modality imaging, augmented reality, and optical coherence tomography are commonly used [4]. However, most works and studies are currently focused on fluorescence and fluorescent dyes. Both, open and mini-invasive type of partial nephrectomy can be enhanced by fluorescent dyes [5, 6].

The first use of fluorescence in medicine was performed by Roger Moore in 1947. He published the usage of fluorescein in Science journal [7]. In 1948, Moore published the results of 46 patients with mixed intracranial tumors. He injected the fluorescent dye intraoperatively into the tumor and correctly identified 44 (96%) malignant tumors [8]. Protoporphyrin IX, hypericin, fluorescein, and indocyanine green (ICG) are fluorescent dyes which are used in urology to facilitate surgical resection in various forms [9]. The most widely adopted fluorescent dye in urologic surgery is ICG.

ICG is a dye in which the light of a wavelength of 803 nm provokes a detectable emission of photons with a wavelength of 820–830 nm after reaching the ICG molecule. This emission is detected by using a high-resolution camera, and pseudocolor software transforms it into a green-colored picture [10]. ICG was invented by Kodak Photography company in 1955 [11], received an FDA approval in 1959 [12], and nowadays it belongs among the most researched fluorescent dyes. ICG is eliminated from blood circulation exclusively by liver cells and completely secreted to the bile. Reactions are catalysed by glutathione s-transferases [13]. ICG is a safe substance. Adverse events were described in 4 of over 240,000 intravenous administrations (including urticaria, severe hypotension, and anaphylactic reaction) [14–16]. Nowadays, ICG is used in colorectal surgery, gynaecology, ophthalmology, dermatology, and cardiology during angiography [17–20]. In the field of urology, ICG can be applied during open, laparoscopic, and robotic surgeries in both oncological and nononcological diagnoses [5, 21]. However, main attention belongs to malignant diseases, especially renal and prostate cancer.

Our work presents description and outcomes of 37 patients with renal tumor who underwent robotic partial nephrectomy enhanced by usage of fluorescent dye - ICG. Nowadays, ICG is not a part of standardly recommended renal tumor treatment.

## 2. Materials and Methods

### 2.1. Fluorescence Imaging

Fluorescence imaging and visualization of fluorescent dyes work due to the standard principle of fluorescence: nonthermal light stimulates the excitation of molecules in substance, capable of fluorescence. Due to this phenomenon, free electrons are activated and moved into an excited position. However, this state is unstable and excited electrons return to the starting position. This return is accompanied by the emission of light energy of wavelength higher than the wavelength of the light that induced the fluorescence [10]. High-resolution digital imaging systems, such as Firefly® imaging mode in the daVinci SI HD® surgical system, can capture this emitted light energy by a high-resolution camera and transfer it into a pseudocolored image as the output. The Firefly® imaging system was approved by the United States Food and Drug Administration for the visible and near-infrared fluorescence endoscopic visualization of vessels, blood flow, and related tissue perfusion in February 2011 [22].

### 2.2. Patients

**Figure 1:**
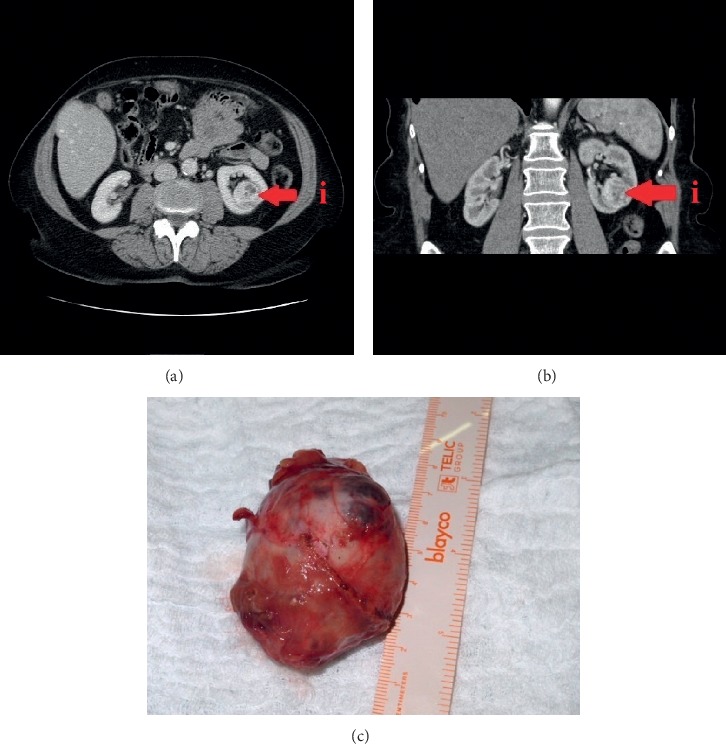
(a) Preoperative CT imaging of (i) endophytic renal tumor before partial nephrectomy in axial projection. (b) Preoperative CT imaging of (i) endophytic renal tumor before partial nephrectomy in coronary projection. (c) Excised renal tumor after partial nephrectomy.

A total of 37 patients with renal cancer underwent robotic partial nephrectomy with intravenous application of ICG between April 2015 and May 2019. Twenty-five men and 12 women participated in the study. Elevated creatinine preoperatively was measured in 2 patients. ICG was used by the surgeon's decision according to unfavourable anatomical features of tumor or to high R.E.N.A.L. nephrometry score or in cases where the endophytic growth of tumor was found [23, 24]. An average diameter of tumor was 31 ± 12 mm, measured on preoperative CT examination (see Figures 1(a) and 1(b)).

Results were evaluated retrospectively. Demography, tumor complexity, and pathological data of 37 patients are summarized in Table 1.

### 2.3. Robotic Partial Nephrectomy

Robotic partial nephrectomy is initiated with the patient in the supine position, under general endotracheal anaesthesia. The patient is placed in the modified flank position. A high flow, low pressure pneumoperitoneum is achieved by using a Veress needle. An incision is made superolateral to the umbilicus (pararectal) for the 12 mm camera port. Under visualization, two 8 mm robotic ports are placed superior and inferior in the medioclavicular line. Additional 12 mm port is placed below the two 8 mm robotic ports in the midline, superior or inferior to the umbilicus. After preparing a position of patient on his contralateral side and after placing robotic ports, the daVinci SI HD® surgical system with the near-infrared fluorescence imaging mode is docked. Prior to renal tumor dissection, the renal hilus (renal artery and vein) is exposed. The renal vein/artery is dissected by a combination of blunt dissection and electrocautery. Surgical dissection should proceed slowly to avoid vascular injury and blood loss. After the preparation of the renal hilus, the tumor and its feeding vessels are dissected and the bulldog clamp is precisely positioned on the arterial branch, selected by the surgeon. Then, freshly prepared ICG is injected intravenously by an anaesthesiologist. The image of nonperfused tissue and exact tumor's area is observed. This area of tumor is precisely margined and excised using mainly cold scissors. In necessary cases, electrocautery is used minimally. Tumorous tissue is removed with attached perirenal fat to achieve complete tumor resection and to enable an optimal pathologic examination of the surgical margins afterward. Running suture over defect is performed to enclose the margins and to reduce the haemorrhage. Then, the surgeon removes the bulldog clamp, performs precise suturing of visible bleeding, and repairs renal calices, if necessary. The local haemostatic agent Surgicel® is applied into the tumor bed, and renorrhaphy is performed with the sliding clip technique. The tumor is removed from the patient's body in an extraction bag (see Figure 1(c)).

### 2.4. ICG Administration

**Figure 2:**
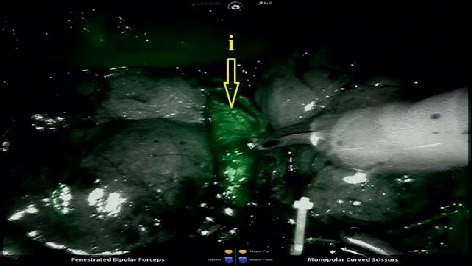
Pass of ICG through (i) one of the main branches of the renal artery after ICG administration.

ICG (VERDYE®) is prepared according to the instruction of producer: 25 mg of ICG dissolved in 5 mL of distilled water. The ICG solution of final concentration of 5 mg/mL is obtained. Then, the anaesthesiologist applies a total amount of 5 mg of freshly prepared ICG intravenously. After the injection of ICG solution, the Firefly™ technology is activated and the light is switched to near-infrared fluorescent light. The pass of ICG is observed as green-light areas on the image in the course of main renal artery (see Figure 2) and subsequently in the course of renal vein after approximately 75 seconds.

**Figure 3:**
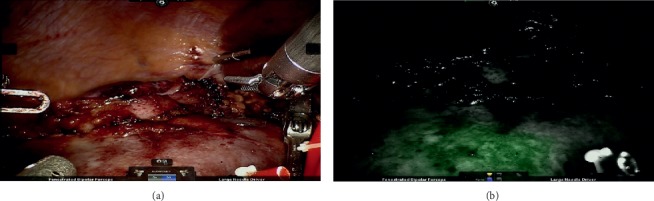
(a) Renal tissue before application of ICG. (b) Borderline between perfused and nonperfused renal tissue after ICG administration with selective arterial clamping.

An exact discrimination and border between perfused and nonperfused tissue can be detected (see Figure 3).

**Figure 4:**
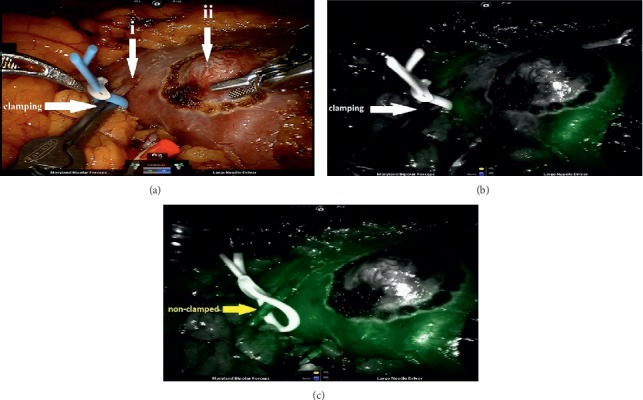
(a) Imaging of (ii) renal tumor and it's (i) feeding artery branch before application of ICG. (b) Perfusion of renal tumor and the area near to renal tumor with superselective arterial clamping after ICG injection. (c) The same renal tissue with ICG-imagined perfusion without superselective arterial clamping.

It allows the surgeon to replace the bulldog clamp to the exact tumor's branch of the renal artery to reduce the blood loss and the ischemia of health renal tissue at the same time (see Figure 4). No adverse events during usage of ICG were noticed.

## 3. Results

Results are summarized in Table 1. Robotic partial nephrectomy with ICG-fluorescence imaging navigation was performed in 29 cases staged cT1a and in 8 cases staged cT1b. A total of 76% cases showed a tumor in the moderate risk group according to the R.E.N.A.L. nephrometry score system. The preoperative average diameter of tumor was 31 mm, the mean surgery time was 133 minutes, the mean estimated blood loss was 190 mL. In cases where arterial clamping was performed (35 cases), the mean duration of warm ischemia of the immediate surrounding area was 14 minutes. Application of ICG revealed specific tumor-supplying vessel in 25 cases (68%), where superselective clamping was performed. According to the Clavien-Dindo classification, two (5%) complications grade II (excessive bleeding) occurred intraoperatively. Two blood transfusions in both cases were applied. One case (3%) developed high-grade complication (grade III) when the diaphragm was damaged by surgical instrument with the necessity of resuturing. There were no intraoperative complications of grade IV and V. Renal function changes (creatinine and glomerular filtration rate (GFR)) before the patient's discharging from hospital showed favourable results for the cases with superselective clamping. Totally 2 (5%) patients were discharged with significantly elevated value of creatinine comparing to preoperative values. Positive surgical margins were detected in 3 (8%) cases. The majority of tumors were found to be malignant on final histopathological results: 28 (76%) tumors were described as clear cell renal cell carcinoma, 1 (3%) as cystic clear cell renal cell carcinoma, and 2 (5%) as papillary renal cell carcinoma. In 3 (8%) cases, histopathological report showed an angiomyolipoma, 3 (8%) cases were verified as an oncocytoma.

## 4. Discussion

To the best of our knowledge, this is the first work which describes results of usage of ICG during robotic partial nephrectomy in greater group of patients with renal tumors in the Czech Republic.

Brassetti et al. defined main criteria for successful partial nephrectomy (trifecta score) as negative surgical margins, no intraoperative complications, and minimal loss of renal function [3]. Cacciamani et al. published extensive meta-analysis of 98 articles, evaluating also the operative, intraoperative, functional, and oncological outcomes of robotic partial nephrectomies. They reported the rate of positive surgical margins up to 13% and the mean number of intraoperative complications up to 20%, respectively [25]. Our work presents similar results: positive surgical margins in 8% and occurrence of intraoperative complications in 8% of all cases, respectively.

We noticed the reduction of renal function in 5% of all patients in our study. Except of trifecta score, the mean warm ischemia time is another important parameter for successful partial nephrectomy and subsequently for preservation of renal function (when clamping is performed). Froghi et al. published a meta-analysis, where they quoted 16 nonrandomized observational studies included 701 patients who underwent robotic partial nephrectomy. They specified the mean warm ischemia time between 18 and 32 minutes [26]. Our study reports the mean warm ischemia time as 14 minutes.

Several reviews compared off-clamp partial nephrectomies with conventional on-clamp surgeries with conflicting results [27, 28]. Simone et al. published comprehensive work showing off-clamp partial nephrectomy was associated with increased intraoperative blood loss and perioperative transfusion rates compared to on-clamp partial nephrectomy. Review was limited by the lack of prospective randomized trials [29]. Moreover, they showed the off-clamp partial nephrectomy group had significantly higher probabilities of maintaining unmodified GFR after surgery in the first 8 years of follow-up comparing with patients undergoing the on-clamp technique [30]. Our study consists mostly of on-clamp partial nephrectomies (clamping or superselective clamping). Number of patients in the off-clamp group was significantly lower. Therefore, statistical evaluation was not performed.

There are several types of ICG usage during partial nephrectomy. First one is based on imaging of arterial perfusion of kidney after intravenous ICG administration. ICG makes bond with plasma proteins rapidly after administration. Thanks to this phenomenon, the branches of main renal artery can be identified and selectively clamped to reduce the damage of normal renal parenchyma due to ischemia [21]. The second type of using ICG during partial nephrectomy, which is not the subject of our work, is based on the feature of ICG to make chemical connection with transmembrane protein, called bilitranslocase (BLT), which transports it intracellularly. BLT is found in high concentrations in the renal proximal and distal convoluted tubules [31]. Thus, normal renal parenchyma emits fluorescent light after ICG administration. Cells of renal cell carcinoma do not express BLT and do not store ICG intracellularly. This makes a different picture of tumor and health parenchyma under near-infrared light (tumor tissue is darker due to its hypofluorescence). However, this imaging depends on overdosing of ICG [21]. Finally, the third and novel technique of ICG usage for totally endophytic robotic partial nephrectomy is using a lipiodol-ICG mixture, delivered into arterial branches feeding the renal mass prior to transperitoneal off-clamp partial nephrectomy. The principle is based on preoperative angiography, where the ICG-lipiodol mixture is selectively delivered into the tertiary-order arteries. Lipiodol serves to avoid a rapid ICG washout from the renal tumor [32].

Among the first pioneering works on the field of ICG-enhanced partial nephrectomy, Tobis et al. administered ICG intravenously to 15 patients who underwent open partial nephrectomy. In 3 patients, ICG clearly identified the renal hilar vessels and guided selective arterial clamping. There was no positive surgical margin [5]. In 2011, Tobis et al. [6] published results of another way of using ICG during partial nephrectomy; they detected renal cell carcinoma through hypofluorescence of cancer cells due to lack of BLT in their membranes. Resected tissues were sent to histopathological examination. Of the 11 patients, 10 demonstrated malignancy on final histopathology. Of the malignant tumors, 7 were hypofluorescent and 3 were isofluorescent compared to the surrounding renal parenchyma with near-infrared fluorescence [6].

In 2012, Krane et al. compared results of 47 patients who underwent robotic partial nephrectomy, enhanced by ICG, with 47 patients treated by robotic partial nephrectomy without usage of ICG. This was the first prospective study with ICG. Warm ischemia time was significantly decreased in the ICG group even with the use of global ischemia. Positive margin rate was similar in both groups, which indicates that ICG does not provide a more precise excision of tumor [33].

Studies with off-clamp partial nephrectomy have shown a significantly higher postoperative GFR after 6 months compared to partial nephrectomies where the renal artery was clamped [34, 35]. Borofsky et al. described superselective renal artery clamping in 27 patients who underwent partial nephrectomy enhanced by ICG. The healthy renal parenchyma was saved of ischemia damage thanks to this superselective clamping. The maximum loss of glomerular filtration in 3 months after surgery was 1.8% vs. 14.9% in the non-ICG group. It shows eventuality of replacing the off-clamp technique in partial nephrectomy by the selective clamping technique using ICG for reduction of postoperative GFR deprivation [36].

Among the Czech researchers and authors, Kočárek et al. presented the administration of ICG with selective arterial clamping during partial nephrectomy. ICG was diluted in distilled water to a concentration of 2.5 mg/mL. They injected this freshly prepared solution intravenously in a total amount of 1.5 mL (3.75 mg of ICG), followed by successful identification of nonperfused tumorous tissue [37].

ICG can be conjugated with specific molecule or radiolabel to improve tissue imaging in approaches known as tumor-targeted imaging and dual-modality imaging. Muselaers et al. described probably the most suitable antigen to target clear cell renal cell carcinoma: carbonic anhydrase IX (CAIX) and its monoclonal antibody Girentuximab. Tagged with fluorescent dye, this tracer can be used as a probe for ccRCCs and accurate tumor delineation during surgery. Labelled Girentuximab can be used for clear cell type of renal cell carcinoma only. Other types of RCC do not express enough amount of CAIX to target [38]. Visualization of tumor by conjugation of fluorescent dye and tumor-specific molecule is limited for superficially localised tumors only. It is caused by limited tissue penetration depth of the near-infrared signal [39]. On the other side, gamma radiation penetrates the tissue deeply. Therefore, tumor-specific molecule can be traced by both: a fluorescent dye and a radiolabel. Tumors are localized with a gamma probe and with a fluorescence camera in the same time. A dual-labelled probe has the advantage that the same tracer can be used for preoperative (positron emission tomography/computed tomography or single photon emission computed tomography/computed tomography (SPECT/CT)) and intraoperative imaging. This fusion of pre- and intraoperative imaging helps to improve the surgeon's orientation [40]. Minn et al. performed in vitro and in vivo evaluation of a dual-labelled ligand, [^64^Cu]XYIMSR-06, for imaging CAIX expression on clear cell renal cell carcinoma by positron emission tomography. In this preclinical study, results indicated pharmacokinetic properties of [^64^Cu]XYIMSR-06 suitable for clinical imaging and superiority of [^64^Cu]XYIMSR-06 comparing with previously reported imaging agents binding to CAIX [41].

## 5. Conclusions

Robotic partial nephrectomy enhanced by ICG administration and subsequent fluorescence imaging is a technically feasible and safe nephron-sparing method of renal cancer treatment with no adverse effects of ICG. Administration of ICG eases superselective clamping of the tumor-specific branch of the renal artery and significantly reduces warm ischemia time of health renal parenchyma which may lead to superior renal function and creatinine and GFR value preservation with favourable surgical quality and oncological results at the same time.

## Figures and Tables

**Table 1:** Demographic, tumor and pathological data, and perioperative outcomes in 37 cases.

Variable	Result
Patients (*n*)	37
Sex (*n*, %)	
Male	25 (68)
Female	12 (32)
Age (years)	
Mean ± SD	57 ± 13
Median (range)	57 (32–79)
BMI (kg/m^2^)	
Mean ± SD	29.0 ± 5.0
Median (range)	28.0 (21.8–48.9)
R.E.N.A.L. nephrometry score (*n*, %)	
Low (4–6)	8 (21)
Moderate (7–9)	28 (76)
High (≥10)	1 (3)
CT tumor diameter (mm)	
Mean ± SD	31 ± 12
Median (range)	28 (13–62)
Estimated blood loss (mL)	
Mean ± SD	190 ± 330
Median (range)	100 (50–1500)
Warm ischemia time (min)	
Mean ± SD	14 ± 5
Median (range)	15 (7–28)
Histopathologic findings (*n*, %)	
Angiomyolipoma	3 (8)
Oncocytoma	3 (8)
Cystic clear cell RCC	1 (3)
Clear cell RCC	28 (76)
Papillary RCC	2 (5)
Surgery time (min)	
Mean ± SD	133 ± 35
Median (range)	120 (70–235)
Positive surgical margins (*n*, %)	3 (8)
Complications (*n*, %)	3 (8)
Minor (CD I-II)	2 (5)
Major (CD III–V)	1 (3)
Creatinine (*μ*mol/L)	
Mean ± SD	80.7 ± 21.1
Median (range)	72.5 (51.8–123.3)
GFR	
Mean ± SD	1.4 ± 0.3
Median (range)	1.5 (0.7–2.0)

BMI, body mass index; CD, Clavien–Dindo; CT, computed tomography; GFR, glomerular filtration rate; RCC, renal cell carcinoma. Data presented as mean ± standard deviation, numbers, with percentages in parentheses or median and range.

## Data Availability

The experimental data used to support the findings of this study are available from the corresponding author upon request.
